# Chitosan-Modified PLGA Nanoparticles for Control-Released Drug Delivery

**DOI:** 10.3390/polym11020304

**Published:** 2019-02-12

**Authors:** Boting Lu, Xikun Lv, Yuan Le

**Affiliations:** State Key Laboratory of Organic-Inorganic Composites, Beijing University of Chemical Technology, Beijing 100029, China; 2016200044@mail.buct.edu.cn (B.L.); lvmhao@163.com (X.L.)

**Keywords:** chitosan, PLGA nanoparticles, paclitaxel, drug release

## Abstract

Poly (lactic-*co*-glycolic acid) nanoparticles (PLGA NPs) are well recognized as an ideal drug delivery carrier for their biocompatibility and biodegradability. In order to overcome the disadvantage of drug burst release, chitosan (CS) was used to modify the PLGA nanoparticles. In this work, CS-PLGA nanoparticles with different ratio of CS to PLGA were prepared using high-gravity rotating packed bed (RPB). With the increase of amount of CS, the particle size increased from 132.8 ± 1.5 nm to 172.7 ± 3.2 nm, zeta potential increased from −20.8 ± 1.1 mV to 25.6 ± 0.6 mV, and drug encapsulation efficiency increased from 65.8% to 87.1%. The initial burst release of PLGA NPs reduced after being modified by CS, and the cumulative release was 66.9%, 41.9%, 23.8%, and 14.3%, after 2 h, respectively. The drug release of CS-modified PLGA NPs was faster at pH5.5 than that at pH 7.4. The cellular uptake of CS-modified PLGA NPs increased compared with PLGA NPs, while cell viability was reduced. In conclusion, these results indicated that CS-modified, PTX-loaded PLGA NPs have the advantages of sustained drug release and enhanced drug toxicity, suggesting that CS-modified NPs can be used as carriers of anticancer drugs.

## 1. Introduction

Conventional chemotherapeutic drugs are limited by their toxicity to normal tissues, short circulatory half-life in plasma, limited water solubility, and non-selective therapeutic effects. In the past two decades, nanoparticles (NPs) have attracted considerable interest in the delivery of anticancer drugs and have become an important area of cancer nanotechnology research [1]. Poly (lactic-*co*-glycolic acid) (PLGA) is an FDA-approved polymer with great biocompatibility and biodegradability. Many researches have demonstrated the great potential of PLGA as a carrier for cancer treatment [2,3]. However, one of the major drawbacks of PLGA NPs is that they cannot specifically interact with cells or proteins, which causes an inability to accumulate drugs in target tissues [4,5]. Another disadvantage of PLGA NPs is the presence of drug burst release, which could result in side effects [6,7].

To overcome these limitations, chitosan (CS) was used to modify PLGA nanoparticles. CS is a natural cationic polysaccharide with biocompatibility and biodegradability [8]. Chitosan has the capability of forming hydrogen and covalent bonding due to its –OH and –NH_2_ groups. The protonation of amino group at low pH, which makes chitosan macromolecule charged positively, leads to the mucosal adhesion of chitosan [9]. Therefore, it is necessary to consider pH when using CS. Because the pH of each region of the human body is obviously different [10], it is possible for CS delivery system to achieve targeted delivery of drugs [11]. Sara et al. [12] prepared low molecular weight chitosan-coated PLGA NPs and showed enhanced drug delivery in a weak acid environment. Because of the positive zeta potential, the cellular uptake of CS-modified PLGA NPs could be increased [13,14]. However, the suitable amount of CS used for modification has rarely been reported. Considering these reports, we were interested in how much the amount of CS modification affects the cytotoxicity and cellular uptake of tumor cells. Knowing this, we could more effectively deliver anticancer drugs.

In this study, PLGA NPs modified with different amount of CS were prepared by the nanoprecipitation. The drug loading, encapsulation efficiency, and in vitro drug release of CS-modified PLGA NPs were studied using paclitaxel (PTX) as a model drug. The effects of CS modification of PLGA NPs on the particle size, zeta potential, and surface morphology were explored. The cytotoxicity and cellular uptake of CS-modified PLGA NPs were studied using MDA-MB-231 cells.

## 2. Materials and Methods

### 2.1. Materials

PLGA (MW = 10,000–20,000, with a lactic acid to glycolic ratio of 75:25) and Nile Red (NR, 98%) were obtained from Yuanye Bio-Technology (Shanghai, China). Chitosan (MW = 100,000–2,000,000, viscosity ≤ 800 mPa·s, deacetylation ≥ 80%) was obtained from Sinopharm Chemical Reagent Co., Ltd. (Shanghai, China). Poloxamer 188 (F68) and Paclitaxel (PTX) were obtained from Ouhe Technology Co., Ltd. (Beijing, China). Acetone and acetic acid were obtained from Beijing Chemical works (Beijing, China). Acetonitrile was obtained from Aladdin (Shanghai, China). 4’,6-diamidino-2-phenylindole (DAPI), dimethyl sulfoxide (DMSO), and 3-(4,5-dimethylthiazol-2-yl)-2,5diphenyl-2-H-tetrazoliumbromide bromide (MTT) were obtained from Sigma-Aldrich (Shanghai, China). All other solvents are of analytical grade and used without further purification.

### 2.2. Preparation of PLGA NPs

**Figure 1:**
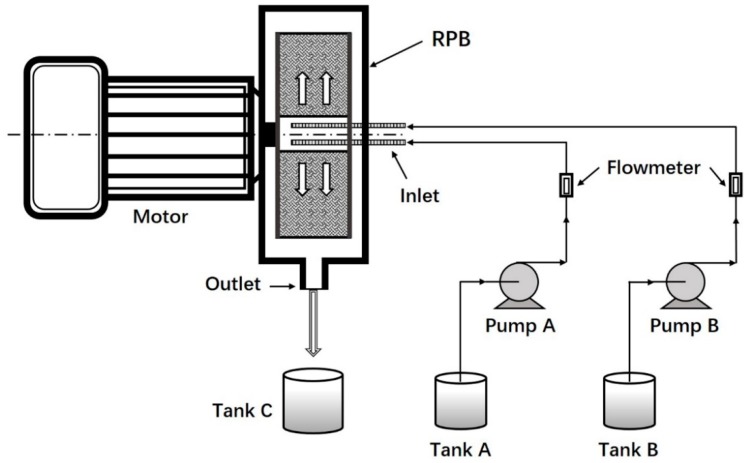
Schematic diagram of experimental setup.

Nanoparticles were prepared by nanoprecipitation using high-gravity rotating packed bed (RPB) reactor (Figure 1). PLGA (200 mg) and paclitaxel (20 mg) were added to acetone (20 mL) to form the organic phase. Poloxamer 188 was added to deionized water (400 mL) to form the aqueous phase (0.3%, w/v). Subsequently, the organic phase with a flow rate of 15 mL/min and the aqueous phase with a flow rate of 300 mL/min were pumped into the RPB reactor. The non-incorporated drug was removed by ultrafiltration centrifugation (12,000 r/min, 15 min). PTX-loaded nanoparticles remained after the supernatant was discarded and the nanoparticles were lyophilized for 48 h (vacuum freeze-drying machine, LGJ-18S, Songyuan Huaxing, Beijing, China).

### 2.3. Preparation of the CS-Modified PLGA NPs

PLGA NPs were modified with CS through electrostatic adherence. Poloxamer 188 (0.3%, w/v), acetic acid (4 mL), and CS (40 mg, 80 mg, 160 mg) were added to deionized water (400 mL) to form the aqueous phase. Other steps are consistent with preparation method of PLGA NPs.

### 2.4. Characterization of the NPs

#### 2.4.1. Particle Size, Zeta Potential, and Surface Morphology of the NPs

The particle size of the NPs was measured by dynamic light scattering (DLS) on Zetasizer (Nano-ZS90, Malvern Panalytical, Malvern, UK). Additionally, zeta potential of the NPs was also measured on Zetasizer. The freshly prepared NPs were diluted with an appropriate amount of distilled water. Each sample was measured three times. Scanning electron microscopy (SEM, JSM-7800F, JEOL, Tokyo, Japan) was used to observe the surface morphology of NPs.

#### 2.4.2. TGA, FTIR, and XPS

The CS content of surface modified NPs was determined by thermal-gravimetric analyzer (TGA, STA-449C, NETZSCH, Selb, Germany). The purified CS or PLGA NPs were placed in alumina crucible and scanned in TGA from 40 to 800 °C at a rate of 10 °C/min under nitrogen flow. The amount of CS in PLGA NPs was determined from the weight loss.

Fourier transform infrared spectroscopy (FTIR, VERTEX 70v, BRUKER, Karlsruhe, Germany) was used to detect the NPs. The dry nanoparticles were weighed at about 2 mg and mixed with the dry potassium bromide (KBr) by grinding. A uniform sheet was made by a tablet press. With air as the background, the wavenumber range was set to 400–4000 cm^−1^, and the resolution was set to 2 cm^−1^.

X-ray photoelectron spectroscopy (XPS, ESCALAB 250, THERMO VG, Waltham, MA, USA) was used to determine the surface element composition of nanoparticles. X-ray source was monochromatic Al Kalph and the power was 150 W. The pass energy was 200 eV for survey and 30 eV for high resolution scans.

#### 2.4.3. Drug Loading and Encapsulation Efficiency

The paclitaxel content in nanoparticles was measured by high performance liquid chromatography (HPLC, MK-II, WATERS, Milford, CT, USA). For HPLC analysis, a reverse-phase XBridge^®^ C18 column (4.6 mm × 150 mm, pore size 5 μm, WATERS, Milford, CT, USA) was used. The mobile phase was a mixture of acetonitrile and phosphoric acid (50:50, v/v), the flow rate was set to 1 mL/min, and the column temperature was set to 30 °C. Paclitaxel was quantified by UV detection at 227 nm. A specific amount of lyophilized nanoparticles was dissolved in 2 mL acetonitrile and then vortexed vigorously for 5 min. Then, the supernatant was collected and filtered with 0.45 μm filter. Drug loading (DL) was the percentage of actual mass of drug loaded in NPs to the total mass of NPs, and the drug encapsulation efficiency (EE) was the percentage of the actual mass of drug loaded in NPs and the initial mass used in the preparation of NPs. The formulas for calculating DL and EE are as follows:

(1) $$\mathrm{DL}=\frac{M}{M_{np}}\times 100\%,$$

(2) $$\mathrm{EE}=\frac{M}{M_{in}}\times 100\%,$$

in which *M* is the actual mass of drug loaded in NPs, *M_np_* is the total mass of NPs, and *M_in_* is the initial mass used in the preparation of NPs.

#### 2.4.4. In Vitro Drug-Release Studies

The in vitro drug release from the NPs was determined in a dissolution tester (Vision G2 Elite 8, HANSON, Chatsworth, CA, USA). 20 mg of PTX-loaded NPs was suspended in 500 mL PBS (pH 7.4 or 5.5) containing 0.5% (w/w) Tween 80 as solubilizer. The rotational speed was set to 100 rpm, and the temperature was set to 37 °C. Sampling was at specified time intervals, and an equal amount of fresh PBS was added. Each drug release experiment was tested in triplicate in vitro. The content of PTX in PBS was then determined by HPLC analysis.

#### 2.4.5. MTT Assay

The cytotoxicity of PTX-loaded NPs was evaluated using the MTT colorimetric assay. The MDA-MB-231 cells were cultured in 96-well plates of Dulbecco’s modified eagle medium (DMEM) containing 10% fetal bovine serum, and the plates were placed in an incubator at 37 °C in an environment of 5% carbon dioxide. The cells were cultured with different concentrations of samples. After the specified incubation time, 20 μL MTT was added to each well, and another 4 h was needed to culture the cells. The medium was then removed and 100 mL DMSO was added to 96-well plates. The color intensity was measured on a microplate reader (Multiskan MK3, THERMO, USA) at 570 nm, and the cell viability was presented and expressed as means ± standard deviation (SD) (*n* = 5).

#### 2.4.6. Cellular Uptake of the NPs

The cells were incubated with neil red-loaded NPs in incubator at 37 °C in an environment of 5% carbon dioxide. After 4 h incubation, the nucleus of cells was stained with DAPI and the cells were fixed with 4% paraformaldehyde at room temperature. The confocal laser scanning microscopy (CLSM, DMi8, LEICA, Wetzlar, Germany) was used to observe the cellular uptake. NR, an organic fluorescent dye, was used to assist in showing the uptake of NPs in tumor cells.

## 3. Results and Discussion

### 3.1. Particle Size, Zeta Potential, and Surface Morphology of the NPs

Particles smaller than 10 nm were quickly eliminated by renal changes, while those larger than 300 nm were removed from the blood circulation due to the recognition of reticuloendothelial system (RES) [15,16]. Therefore, 10 to 200 nm was an ideal range for nanoparticles to promote tumor accumulation. In this study, the particle size of NPs ranged from 132.8 nm to 172.7 nm (Table 1). The particle size increased with the modification amount of chitosan.

The zeta potential of unmodified PLGA NPs was negative (−20.8 ± 1.1 mV), because of the carboxyl end groups of PLGA molecules located on the surfaces of NPs. The zeta potentials of the CS-modified PLGA NPs were positive, as shown in Table 1, indicating that there some of the amino groups of the CS molecule were located on the surfaces of the NPs. Taken together, these results indicated that the CS had been successfully coated on PLGA NPs. The surface charge properties of nanoparticles were related to their stability and cell adhesion properties [17]. Generally, the greater the absolute zeta potential of nanoparticles, the higher the stability in vitro. When the ratio of chitosan to PLGA is more than 0.4, CS-modified PLGA NPs may have higher stability in vitro. In addition, CS-modified PLGA NPs could interact with the negatively charged cell membrane through ionic adsorption [6,12,18], which could contribute to higher cellular uptake.

**Figure 2:**
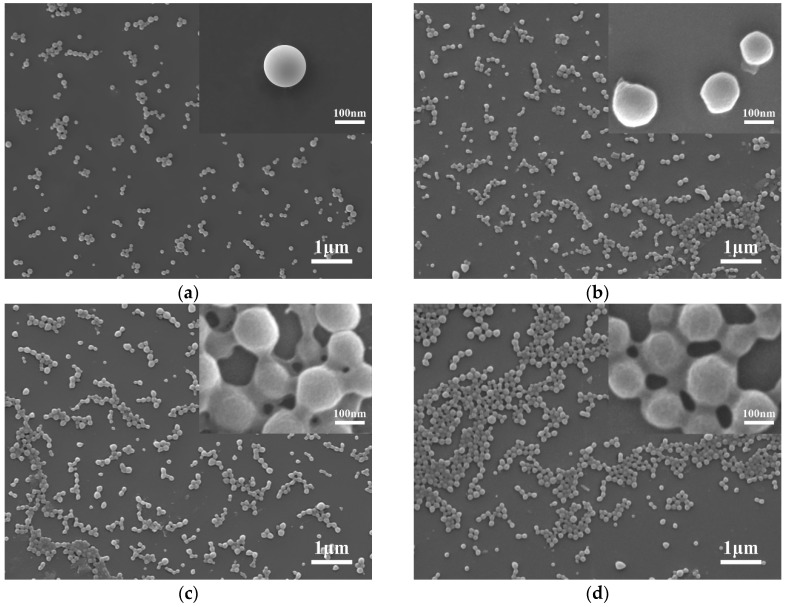
Scanning electron microscopy (SEM) images of the NPs: (**a**) poly (lactic-*co*-glycolic acid) (PLGA); (**b**) chitosan (CS)/PLGA = 0.2; (**c**) CS/PLGA = 0.4; (**d**) CS/PLGA = 0.8.

Surface morphology of the NPs was observed as being smoothly spherical in form using SEM. The CS-modified PLGA NPs were 100–150 nm in size (Figure 2). SEM images of the nanoparticles suggested they were slightly smaller than those measured by DLS measurements (Table 1). This could mean that SEM shows the NPs in a dry state, while DLS method displays the hydrated layers [19]. Due to the interaction between chitosan molecules, it could be seen that there was more obvious adhesion between nanoparticles as the increase of the amount of CS on the surface of nanoparticles. Additionally, the surface of PLGA NPs that was not modified by CS was smoother than the surface of CS-modified PLGA NPs.

### 3.2. TGA, FT-IR, and XPS

**Figure 3:**
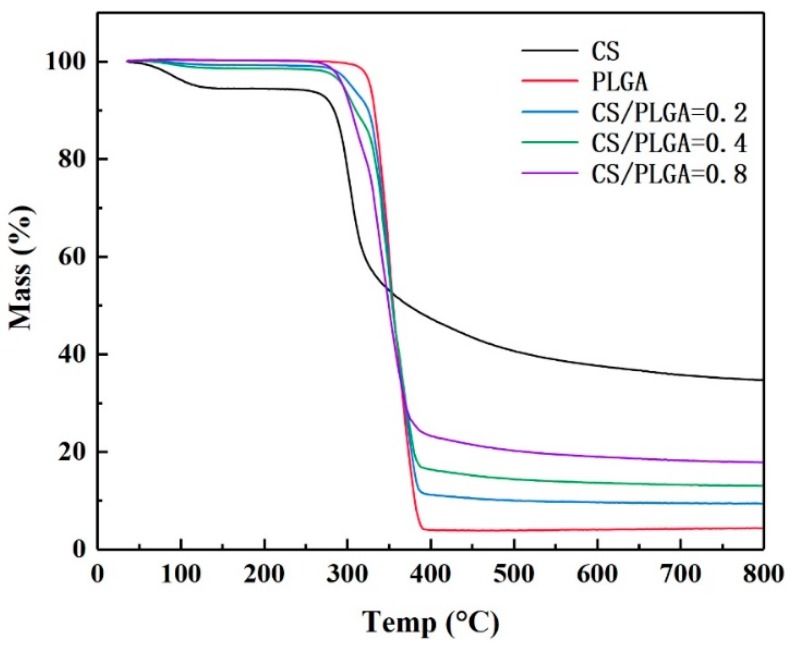
Thermal gravimetric analyzer (TGA) thermograms of NPs.

TGA was used to estimate the CS contents on the surfaces of the NPs (Figure 3). The initial temperature of thermal degradation of PLGA was about 315 °C and degraded completely at 400 °C, with 4.5% remaining. The weight loss of CS can be divided into two stages; the first stage was the stage of losing water. At the beginning of thermal degradation, CS lost a small amount of weight (5%), which indicated that CS contained about 5% free water. The second stage was thermal degradation. CS degraded at about 295°C, and 34.7% still remained at 800 °C. The CS-modified PLGA NPs (CS/PLGA = 0.2, CS/PLGA = 0.4 and CS/PLGA = 0.8) remained 9.4%, 13.0% and 17.8% at 800 °C, respectively. According to the results of thermal analysis, the mass ratios of CS to PLGA were estimated at 19.4%, 39.2%, and 78.7%, respectively, very close to the initio ratio.

**Figure 4:**
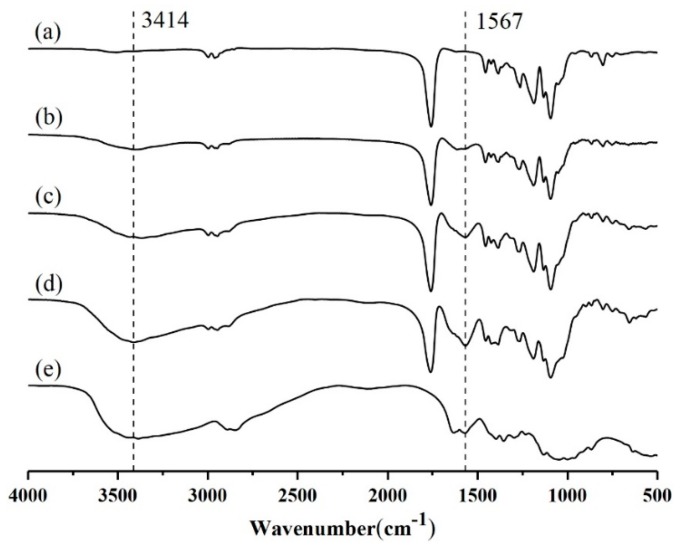
FTIR spectra: (**a**) PLGA; (**b**) CS/PLGA = 0.2; (**c**) CS/PLGA = 0.4; (**d**) CS/PLGA = 0.8; (**e**) CS.

FT-IR spectra proved the existence of CS on PLGA surface (Figure 4). The FT-IR spectrum of PLGA contained a strong peak at 1094 cm^−1^ for C–O–C stretching, as well as a peak at 1758 cm^−1^ for C=O stretching. The characteristic band at 3414 cm^−1^ was ascribed to stretching vibration of –NH_2_ and –OH groups in CS. Due to the existence of amide group and C–H stretching in CS, strong peaks also appeared at 1567 cm^−1^. The characteristic peaks of CS were observed in the FT-IR spectrum of CS-modified PLGA nanoparticles, which indicated that CS successfully modified PLGA nanoparticles. It is noteworthy that there are no new peaks in the FTIR spectra of CS-modified PLGA NPs, or in the disappearance of the original peaks. The FTIR spectra of CS-modified PLGA NPs seemed to be the superposition of spectra of PLGA and CS, which indicated that CS modified PLGA nanoparticles by physical adsorption [17].

**Figure 5:**
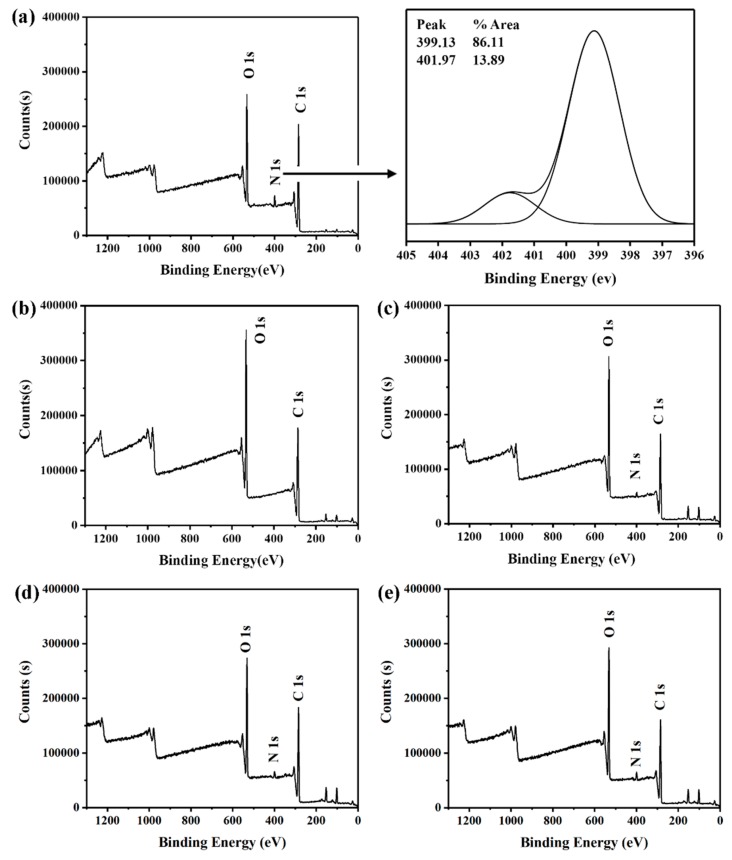
X-ray photoelectron spectroscopy (XPS) spectra: (**a**) CS; (**b**) PLGA; (**c**) CS/PLGA = 0.2; (**d**) CS/PLGA = 0.4; (**e**) CS/PLGA = 0.8.

XPS was a surface element analysis technology, which could provide quantitative and qualitative information for the existence of various elements on the surface of nanoparticles. Therefore, whether CS was modified on the surface of PLGA NPs could be further confirmed by XPS. The binding energy of –NH_2_ was about 399 eV, while that of –NHCO was about 402 eV, corresponding to amine and amide, respectively [20]. As shown in Figure 5, the CS-modified PLGA NPs show the signal of nitrogen near 400 eV, while the PLGA NPs do not observe the signal of nitrogen under the same conditions, which proved that CS has been modified to the surface of nanoparticles.

Furthermore, the N1s signal increased due to the increase of CS. The surface atomic concentration of NPs was listed in Table 2. There was an increase of nitrogen atom concentration as chitosan modification increased. This indicated that there were more amino groups on the surface of CS/PLGA (w/w) = 0.8, compared to CS/PLGA (w/w) = 0.4 and CS/PLGA (w/w) = 0.2. This change also corresponded to the change of surface zeta potential of nanoparticles.

### 3.3. Drug Loading and Encapsulation Efficiency

As shown in Table 3, the EE value of the PLGA NPs without modification was 65.8%. Meanwhile, the EE values of CS-modified PLGA NPs (CS/PLGA = 0.2, CS/PLGA = 0.4 and CS/PLGA = 0.8) were 80.5%, 85.3%, and 87.1%, respectively. Nanoparticles were prepared by the nanoprecipitation method, which was usually used to encapsulate hydrophobic drugs. Drugs and PLGA dissolve in acetone together. When organic phase contacts water phase, solvent displacement occurs at the interface of two phases. Then, drugs and PLGA deposit into nanoparticles at the interface [21,22,23]. The formation of nanoparticles occurs at the interface between two phases, and some drug molecules inevitably diffuse into the aqueous phase, resulting in low encapsulation efficiency. This difference in EE values was attributed to the fact that CS can effectively reduce PTX leakage from NPs. Moreover, CS could effectively adsorb PTX because CS was positively charged and PTX was negatively charged. However, with the change of modification amount of CS, the trend of DL was different from that of EE. Although the modification of CS improved the efficiency of drug loading, the total mass of NPs increased. As a result, the DL value decreased. Overall, the modification of CS still had a positive effect.

### 3.4. In Vitro Drug-Release Behavior of NPs

**Figure 6:**
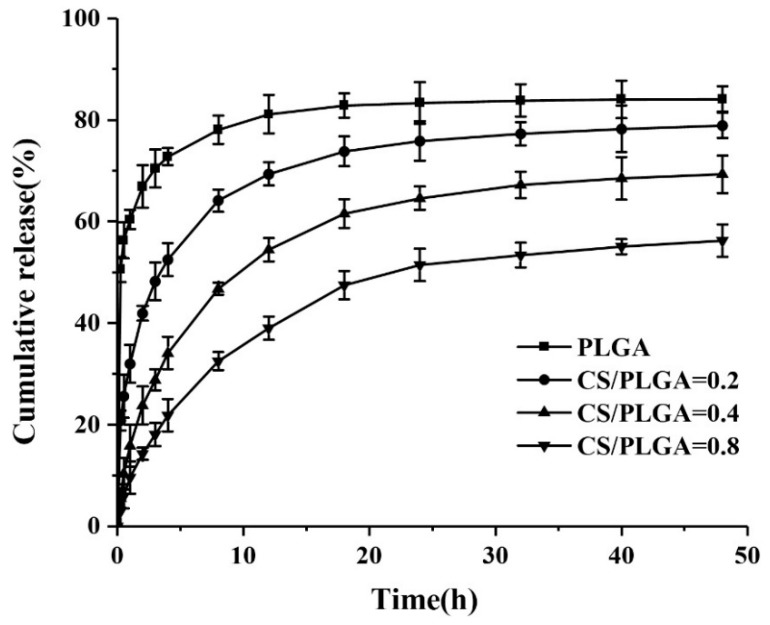
In vitro release of paclitaxel (PTX) from NPs at pH = 7.4.

One of the objectives of this study was to explore the effect of chitosan modification on drug release behavior of nanoparticles. The PBS at pH 7.4 (simulating the pH of normal human blood) was used to study the release of PTX in NPs. The cumulative release curves were showed in Figure 6. The cumulative release of PTX from PLGA NPs reached 66.9% after 2 h because of the presence of burst release. A strong burst release should be avoided, because it can reduce the effect of drug and may cause side effects on the human body [24,25,26,27]. Generally speaking, burst release always exists in in vitro drug release of nanoparticles prepared by nanoprecipitation, and it was caused by the adsorption of drugs on the surface of nanoparticles [23]. Charge attraction between drugs and chitosan played an important role in sustained drug release [6]. Due to the adsorption of drugs on chitosan on the surface of nanoparticles, the leakage of PTX was reduced, and the initial release rate of PTX in CS-modified PLGA NPs was significantly slower than that in PLGA NPs. Moreover, the larger the chitosan content in the NPs, the slower the initial release rate of PTX. The cumulative release of PTX from CS-modified PLGA NPs was 41.9%, 23.8%, and 14.3%, after 2 h, respectively. CS-modified PLGA NPs showed more sustained release after the initial rapid release. This result showed that surface modification of CS on PLGA NPs can effectively solve the problem of initial burst release of PLGA NPs and achieve more moderate and sustained release.

**Figure 7:**
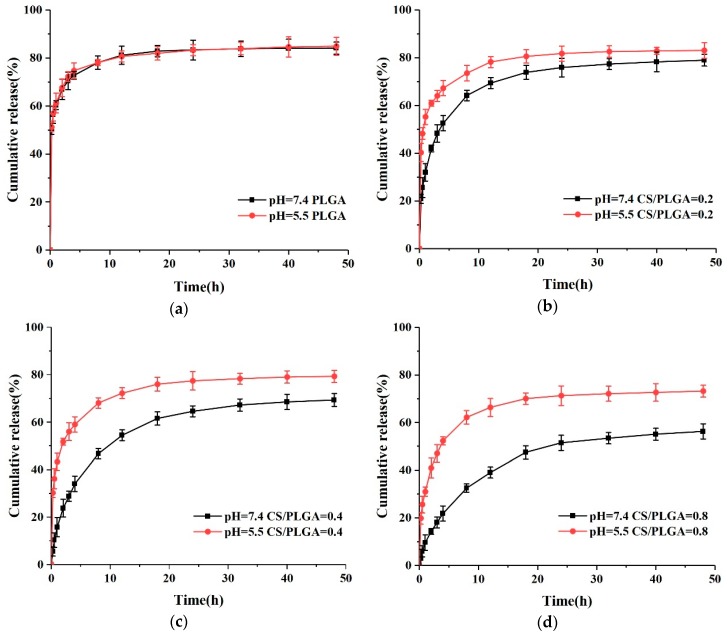
In vitro release of PTX from NPs at pH 7.4 and pH 5.5: (**a**) PLGA; (**b**) CS/PLGA = 0.2; (**c**) CS/PLGA = 0.4; and (**d**) CS/PLGA = 0.8.

The pH-responsive nanoparticles can release drugs rapidly in the tumor site and slowly or even not at all in the peripheral circulation [28,29,30]. The pH-responsive release of nanoparticles was investigated by two different buffer solutions (pH = 5.5 and pH = 7.4) in vitro. As shown in Figure 7a, the release patterns of PLGA NPs at two different pHs were similar, which indicated that the release of PTX-loaded PLGA NPs was pH-independent. However, the release rate of PTX in CS-modified PLGA NPs was very low at pH 7.4, and only 41.9%, 23.8%, and 14.3% PTX was released within 2 h, respectively. Meanwhile, 60.9%, 51.7%, and 40.9% PTX was released after 2 h at pH 5.5, respectively, indicating that CS-modified NPs had pH-responsive properties. The pKa of Chitosan is to 6.3 due to the free amino group [14,20,31]. When the pH is higher than 6.3, these amino groups deprotonate and uncharge, thus forming an insoluble biopolymer shell. However, when the pH is less than 6.3, these amino groups are protonated and the solubility of chitosan in acidic pH increased [31]. Due to the higher solubility of CS at pH 5.5, CS on the surface of NPs could no longer effectively slow down the release rate of PTX.

### 3.5. MTT Assay

**Figure 8:**
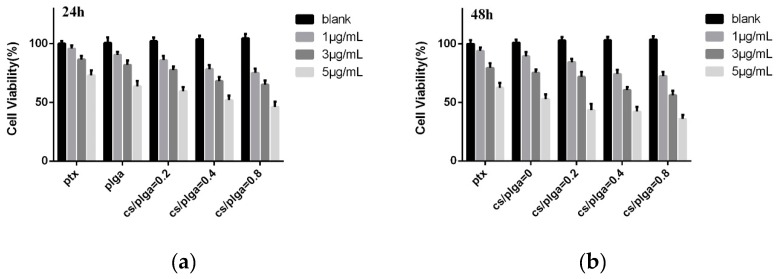
Cell viability of MDA-MB-231 cells: (**a**) 24 h and (**b**) 48 h.

Although the safety of PLGA has been widely recognized due to its biocompatibility and biodegradability, it is possible that CS-modified PLGA NPs were cytotoxic. Blank NPs were also evaluated. Figure 8 showed the effect of PTX-loaded NPs and blank NPs on the proliferation of MDA-MB-231 cells. According to the results, the cell viability of blank NPs increased slightly with the increased amounts of CS coating on the nanoparticle, indicating that CS were not cytotoxic and CS even had a positive effect on the safety of PLGA NPs. Compared with free PTX, the PTX-loaded NPs could reduce the cell viability of MDA-MB-231 cells, which means NPs had a higher level of cytotoxicity. According to previous reports [32], drug-loaded nanoparticles can enter tumor cells through endocytosis and then release the drug into cells. This approach could overcome the resistance mechanism of tumor cells by avoiding the efflux of P-glycoprotein. Meanwhile, the cell viability decreased significantly with the increase of chitosan modification, suggesting that chitosan modification may contribute to the cellular uptake of PLGA NPs.

### 3.6. Cellular Uptake of the NPs

**Figure 9:**
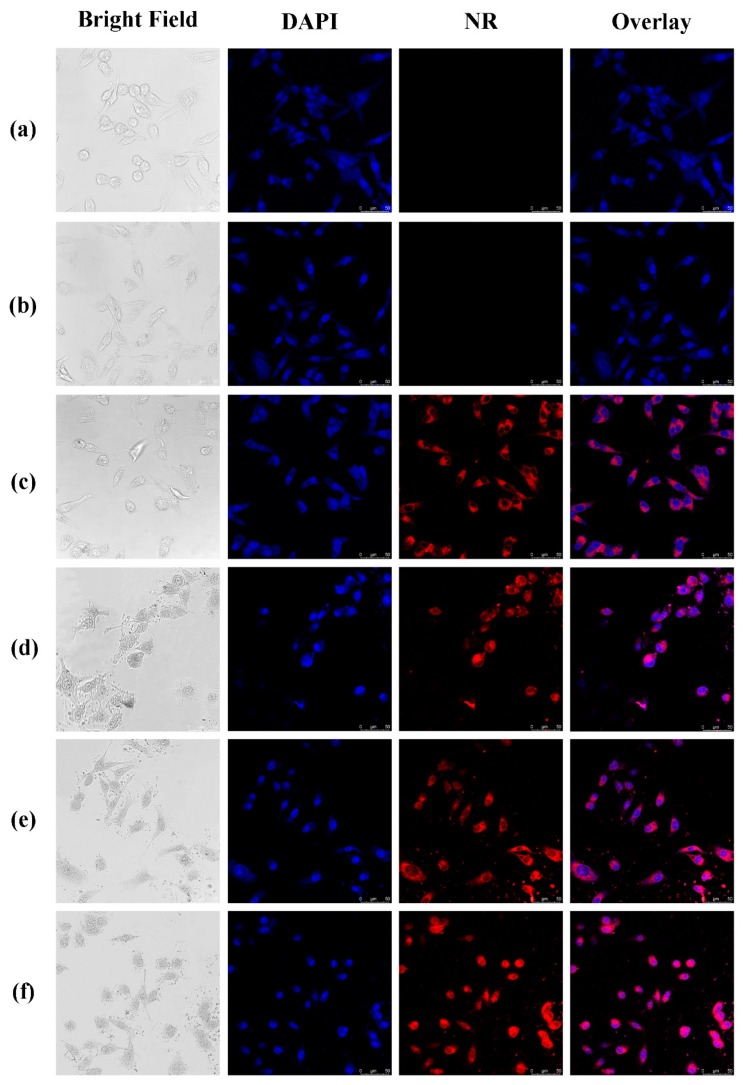
Confocal laser scanning microscopy (CLSM) images: (**a**) control; (**b**) raw NR; (**c**) PLGA; (**d**) CS/PLGA = 0.2; (**e**) CS/PLGA = 0.4; and (**f**) CS/PLGA = 0.8.

CLSM was used to show the uptake of NPs by tumor cells. NR-loaded NPs were prepared by the same method as PTX-loaded NPs. NR was a water-insoluble organic fluorescent dye, and existed in the form of large particles in aqueous solution. As shown in Figure 9, the free NR could not enter MDA-MB-231 cells, and NR-loaded NPs could effectively enter MDA-MB-231 cells through endocytosis. This result demonstrated the advantage of NPs in transporting drugs into cells.

Compared with NR-loaded PLGA NPs, CS-modified PLGA NPs show higher cellular uptake. Positive-charged CS-based NPs can promote endocytosis by interacting with negatively charged cell membranes [33]. Therefore, CS-modified PLGA NPs can enhance drug uptake by tumor cells [34]. It is worth noting that CS-modified PLGA NPs entered the nucleus of MDA-MB-231 cells while PLGA NPs did not. Previous studies [35,36] stated clearly that PLGA nanoparticles could enter the nucleus with the help of surface-modified cationic polymers. According to Figure 9, the amount of modification of CS was an important factor affecting the entry of NPs into the nucleus. Meanwhile, it needed to be large enough to ensure that NPs could enter the nucleus effectively. These results show the potentiality of CS-modified PLGA NPs as gene carrier.

## 4. Conclusions

In this study, PLGA NPs were modified by different amount of CS. The particle size of nanoparticles was in an appropriate range, and changed from 132.8 nm to 172.7 nm. The zeta potential of PLGA NPs became positive after being modified by CS. The efficiency of drug loading increased with the increase of the amount of CS. The initial burst release of PLGA NPs was effectively reduced after being modified by CS, and presented a more sustained release. CS-modified PLGA NPs were pH-responsive and released faster at pH5.5, which contributed to faster drug release in tumor tissue. Meanwhile, CS-modified PLGA NPs showed higher levels of cytotoxicity and cellular uptake in MDA-MB-231 cells than PTX-loaded PLGA NPs and free PTX. These results suggested that the CS-modified PLGA NPs have potential as an antitumor drug carrier. The amount of CS modified on the surface of PLGA NPs could affect the properties of PLGA NPs, and suitable carriers could be designed to deal with this problem.

## Figures and Tables

**Table 1:** Particle size, polydispersity index (PDI), and zeta potential of nanoparticles (NPs).

NPs	Particle Size (nm)	PDI	Zeta Potential (mV)
PLGA	132.8 ± 1.5	0.155 ± 0.03	−20.8 ± 1.1
CS/PLGA (w/w) = 0.2	140.5 ± 2.4	0.104 ± 0.02	10.1 ± 0.9
CS/PLGA (w/w) = 0.4	154.2 ± 2.6	0.122 ± 0.04	21.5 ± 0.5
CS/PLGA (w/w) = 0.8	172.7 ± 3.2	0.144 ± 0.06	25.6 ± 0.6

**Table 2:** Surface atomic concentration of NPs.

NPs	C1s	O1s	N1s
PLGA	64.98	35.02	0
CS	69.03	26.73	4.24
CS/PLGA (w/w) = 0.2	65.02	32.2	2.78
CS/PLGA (w/w) = 0.4	66.29	30.19	3.52
CS/PLGA (w/w) = 0.8	68.21	27.73	4.06

**Table 3:** Drug Loading Efficiency and Encapsulation Efficiency of NPs.

NPs	DL (%)	EE (%)
PLGA	5.07	65.8
CS/PLGA (w/w) = 0.2	6.42	80.5
CS/PLGA (w/w) = 0.4	5.69	85.3
CS/PLGA (w/w) = 0.8	4.59	87.1
